# Rosmarinic acid attenuated inflammation and apoptosis in folic acid-induced renal injury: Role of FoxO3/ NFκB pathway

**DOI:** 10.22038/ijbms.2024.80551.17436

**Published:** 2025

**Authors:** Maryam Mottaghi, Fatemeh Heidari, Tahereh Komeili Movahed, Akram Eidi, Azam Moslehi

**Affiliations:** 1 Department of Biology, Science and Research Branch, Islamic Azad University, Tehran, Iran; 2 Cellular and Molecular Research Center, Qom University of Medical Sciences, Qom, Iran; 3 Department of Physiology and Pharmacology, Faculty of Medicine, Qom University of Medical Sciences, Qom, Iran

**Keywords:** Apoptosis, Folic acid, Inflammation, Kidney injury, Rosmarinic acid

## Abstract

**Objective(s):**

Rosmarinic acid (RA) is a herbal compound with various antioxidant and anti-inflammatory effects. This study aimed to explore the anti-inflammatory and anti-apoptotic properties of RA in folic acid-induced renal injury.

**Materials and Methods:**

Thirty-six male C57/BL6 mice were randomly divided into six groups (N=6): Control (received normal saline), NaHCO_3_ (received NaHCO_3_ as folic acid solvent), FA (received folic acid (FA)(IP) to induce renal injury), RA (received 100 mg/kg RA), RA50-FA (received 50 mg/kg RA solution after folic acid injection), and RA100-FA (received 100 mg/kg RA after folic acid injection). For ten days, the treatment groups received RA by gavage. The effects of RA were assessed using H & E staining, biochemical tests, western blotting, and ELISA in the kidney tissues of the mice. Real-time RT-PCR was also performed to evaluate the expression changes of renal genes.

**Results:**

Our data showed that treatment by RA led to the over-expression of FoxO3 (*P*<0.05) and decrease in NFκB levels (*P*<0.01 and *P*<0.05) and expression of TNFα (*P*<0.05) and IL6 (*P*<0.001 and* P*<0.01). Other evaluations showed a decrease in p53 (*P*<0.01 and *P*<0.001), Bax/Bcl-2 ratio expression (*P*<0.01 and *P*<0.05), and Caspase-3 level (*P*<0.01 and *P*<0.05) compared to the folic acid group. Histological and biochemical results also confirmed the attenuation of tissue damage.

**Conclusion:**

This study revealed that RA’s positive effects on folic acid-induced renal injury might result from the involvement of the FoxO3/NFκB pathway, thereby suppressing inflammation and apoptosis.

## Introduction

Nearly 10% of adults worldwide have been diagnosed with chronic kidney disease (CKD), which is linked to the development of end-stage renal disease (ESKD) and then dialysis or transplantation, hospitalization problems, and early death (1). Renal injuries can be brought on by several risk factors, including ischemia, sepsis, medication toxicity and overdose, exposure to heavy metals, and diabetes (2). The B-group vitamin folic acid is naturally present in green leafy vegetables, citrus fruits, and legumes, as well as in foods derived from animals, such as eggs, and it can reduce the incidence of neural tube defects (NTDs) in the fetus (3). Nevertheless, there are many reports that excessive use can have side effects, such as renal damage (4). Oxidative stress, mitochondrial abnormalities such as bioenergetics and mitophagy-related disorders, pyroptosis, ferroptosis, and elevated production of fibroblast growth factor 23 (FGF23) are the main mechanisms of renal injury induced by FA (5-7).

Chronic inflammation is a distinguishing sign of chronic renal disease, and a rise in inflammatory markers is a hallmark of inflammation in CKD (8). Tumor necrosis factor-alpha (TNF-α) and interleukin-6 (IL-6) are important inflammatory mediators and play a key role in the pathophysiology of CKD (9,10). Caspases and nuclear factor-B (NF- κB) are major pathways that activate TNF-α (11).

Numerous renal illnesses are thought to be caused by inflammation, which can also affect the activation and inhibition of autophagy and eventually disturb cell recycling (12, 13). A cellular recycling process called autophagy involves the self-degradation of damaged organelles and proteins and their repair (14). The pathophysiology of renal disorders revolves around renal cell death (15). The most important transcription factor in the Forkhead box O family, Forkhead box O3a (FoxO3a), is related to cell proliferation, apoptosis, autophagy, oxidative stress, and aging (16). FoxO3 plays an important role in ameliorating inflammation and apoptosis in renal injuries (17). Deacetylated FoxO3 enters the nucleus and changes gene expression, such as IL6, caspase3, and Bcl2 family (18, 19). Proapoptotic proteins such as P53 and Bax are related to apoptotic cell death in kidney disorders, and inhibiting their activity can decrease the development of the condition (20). Also, the reduction of Bcl-2 and the increase of Caspase-3 can help recover renal damage (21). Renal autophagy has been demonstrated to be impaired by ischemia, toxic damage, and inflammation. In addition, dysregulated, excessive, or faulty autophagy can cause chronic inflammation and promote cell death (22).

Considering traditional treatment with anti-inflammatory and antioxidant drugs, growing data suggest several plant-derived minerals, vitamins, and metabolites have therapeutic effects on CKD (8, 23). A polyphenol known as rosmarinic acid (RA) has anti-inflammatory, antioxidant, and neuroprotective effects (23). According to studies on RA’s preventive and antioxidant properties, the kidneys were protected against the early stages of diabetic nephropathy and diethylnitrosamine-induced nephrotoxicity (24, 25). There are also reports of protective and therapeutic effects of RA on acute renal toxicity (26, 27). 

Therefore, the current study aimed to investigate RA’s anti-apoptotic and anti-inflammatory effects on folic acid-induced renal injury.

## Materials and Methods

### Materials

Rosmarinic acid (536954-5G) was purchased from Sigma-Aldrich (St. Louis, MO, USA), and folic acid was acquired from Cayman Chemical (Ann Arbor, MI, USA). Folic acid was set in NaHCO_3_. 

### Animal’s model induction and study protocol

Animal care and experiments were approved by the Institutional Animal Care and Use Committee and conducted following the Guide for Care and Use of Laboratory Animals Health (Ethics code: IR.IAU.SRB.REC.1402.018). Thirty-six male C57/BL6 mice weighing 22–25 g were purchased from the Pasteur Institute in Tehran, Iran. Mice were housed in a 12 hr dark/light cycle animal facility with controlled temperature (20–25 °C) and humidity (40–70%). Food and water were given ad libitum throughout the study.

The animals were adapted to the environment for one week and then randomly divided into six groups of six mice. Control (received normal saline by gavage), NaHCO_3_ (0.2 mL of NaHCO_3_ injection as a vehicle for FA), FA (received a single dose of 250 mg/Kg FA by intraperitoneal injection to induce renal injury) (2), RA (were given 100 mg/kg RA by gavage for ten days) **(**28**)**, RA50-FA (were given 50 mg/kg RA 60 min before Folic acid administration via gavage and continued for ten days) **(**28**)**, and RA100-FA (were given 100 mg/kg RA 60 min before FA administration via gavage and continued for ten days).

Finally, the animals were anesthetized using ketamine (50 mg/kg) and xylazine (0.01 mg/kg). One kidney was fixed in a 10% formalin solution for histopathology assessment, and the other kidney was stored at -80 °C to check cellular evaluations.

### Biochemical analysis

Blood urea nitrogen (BUN) and creatinine (Cr) levels were assayed according to the manufacturer’s instructions (Man Co. kits, Iran). 

### Histopathology evaluations

One of the kidneys was dissected and fixed in a 10% buffered formaldehyde solution. Then, 5μm thick sections from paraffin-embedded renal tissue were prepared for hematoxylin and eosin (H&E) staining. An expert pathologist, blinded to the group definitions, interpreted the histological findings.

### ELISA

Briefly, 100 mg of the renal tissue was weighed, and 1 ml phosphate buffer was added and centrifuged (3000-4000 rpm for 20 min). Supernatants were then collected, allocated, and kept at -80 °C. Caspase-3 and NFκB (Nuclear factor kappa-light-chain-enhancer of activated B cells) were detected in strict line with the ELISA kit instructions (ZB-NFκB-96A, Zellbio company, Germany and ZB-caspase3-96A, Zellbio company, Germany). The optical densities at 450 nm were assessed with a microplate reader (RT-6100, Lei Du).

### Western blot

FoxO3 (Forkhead box O3) expression were determined by western blot. Briefly, the renal tissue protein concentration was measured using a Bradford assay kit from Sigma Aldrich (USA). The proteins were separated and transferred to polyvinylidene fluoride (PVDF) membranes (Roche Diagnostics GmbH, Mannheim, Germany). The membrane was blocked with 5% skim milk for two hours and incubated overnight at 4 °C with primary antibodies against FoxO3(ab23683, Abcam) and β-actin (ab8227, Abcam). After washing, the HRP-conjugated secondary antibody (1:7000, Cell Signaling) was added to the membranes. The membranes were then washed and incubated with enhanced chemiluminescence (ECL, Amersham) reagents in a darkroom. The membrane was exposed to an X-ray film, visualized using the ECL reagent, and detected with the enhanced chemiluminescence detection system (Image Lab™ Touch Software, BIO-RAD, USA). Image J software (version IJ 1.46r, NIH, USA) was used to determine the intensity of the bands, and the relative expression of proteins was normalized to β-actin.

### Real-time RT PCR

Total RNA was isolated from kidney tissue using Trizol, according to the manufacturer’s instructions. RNA concentration was measured by the Nanodrop spectrophotometer (Nanodrop 2000c, Thermos Scientific, USA), although single-strand complementary DNA was synthesized by the cDNA synthesis kit (Yektatajhiz, Iran). 

Real-time PCR reactions were carried out using SYBR Green qPCR Master Mix and specific primers (Table 1). The following protocol was used: initial denaturation for 10 min at 92 °C; 40 cycles at 92 °C for 15 sec, 60 °C for 30 sec, and 72 °C for 30 sec. The .2 $-∆∆C_{t}$method was used to estimate the differences in gene expression. After amplification, the products were verified using a melting curve analysis.GAPDH was considered an internal control.

### Statistics analysis

Statistical analysis was performed using one-way analysis of variance (ANOVA) and Tukey’s test for multiple comparisons using the statistical software SPSS for Windows version 25 (IBM SPSS version 25; USA). Values are expressed as means ± standard error of the mean (SEM). In all tests, *P*<0.05 was considered statistically significant.

## Results

### Effects of RA on renal levels of NFκB and FoxO3 in mice induced by FA

**Figure 1:**
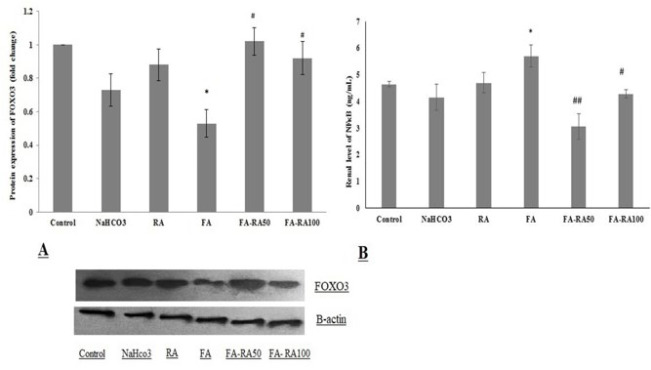
A. Protein expression of FoxO3 in different groups of mice induced by folic acid (Mean ± SEM, N = 6), *P<0.05 compared to the control and RA groups, # *P*<0.05 compared to the FA group. **B.** Renal level of NF-κB in different groups. (Mean ± SEM, N = 6), *P<0.05 compared to the control group, ##*P*<0.01 compared to the FA group, and #*P*<0.05 compared to the FA group (one-way ANOVA followed by Tukey's *post hoc *test)

FoxO3 protein expression markedly declined in the FA group compared to the control group (0.53±0.082 vs 1±0, *P*<0.05), and a significant increase was observed after administration of RA (1.02 ±0.082 and 0.92±0.098 vs 0.53±0.082, *P*<0.01) (Figure 1A). Contrarily, the tissue level of NFκB was significantly higher in the FA group compared to the control group (5.71±0.41 vs 4.65±0.1, *P*<0.05); however, its levels decreased after RA treatment in the FA-RA50 group (3.07±0.48 vs 5.71±0.41, *P*<0.05) (Figure 1B). 

### Effects of RA on renal expressions of IL6 and TNF-α in mice induced by FA

**Figure 2:**
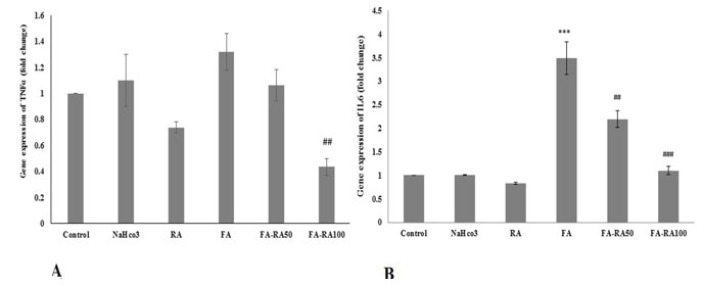
A. mRNA expression of TNFα in different groups of mice induced by folic acid (Mean ± SEM, N = 6), ##*P*<0.01 compared to the FA group. B. mRNA expression of IL6 in different groups. (Mean ± SEM, N = 6), ***P<0.001 compared to the control, NaHCO_3_, and RA groups, ##*P*<0.01 compared to the FA group, and ###*P*<0.001 compared to the FA group (one-way ANOVA followed by Tukey's *post hoc* test)

 As shown in Figure 2A, although in the FA group was no significant overexpression of TNF-α observed compared to the control group, treatment with RA resulted in a significant decrease of TNF-α in the RA100-FA group compared to the FA group (0.43±0.06 vs 1.31±0.14; *P*<0.01). Also, IL6 gene expression markedly rose in the FA group compared to the control group (3.49±0.35 vs 1, *P*<0.001), and both 50 and 100 mg RA administration could significantly deceased it compared to the FA group (2.19±0.18 and 1.09±0.088 vs 3.49±0.35, *P*<0.01 and *P*<0.001, respectively)(Figure 2B).

### Effects of RA on mRNA expressions of P53, Bax/Bcl-2 ratio, and caspase-3 level in mice induced by FA

**Figure 3:**
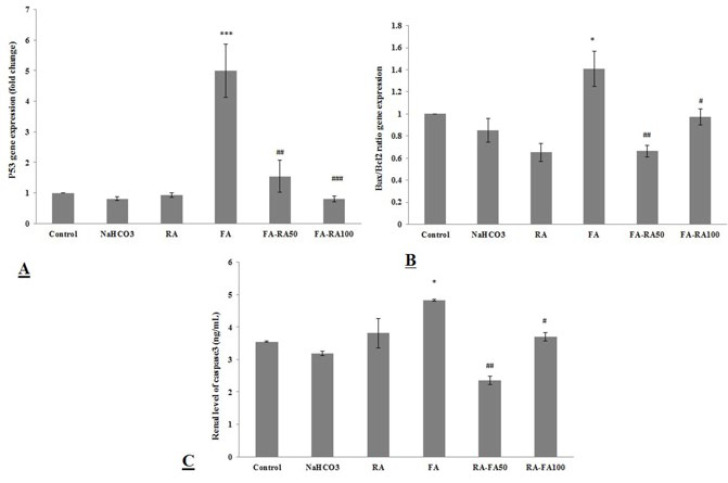
**A.**mRNA expression of P53 in different groups of mice induced by folic acid (Mean ± SEM, N = 6), ***P<0.001 compared to the control, NaHCO_3_ and RA groups, ##*P*<0.01 compared to the FA group and ###*P*<0.001 compared to the FA group. **B.** mRNA expression of Bax/Bcl_2_ ratio in different groups. (Mean ± SEM, N = 6), *P<0.05 compared to the control, NaHCO_3_ and RA groups, ##*P*<0.01 compared to the FA group and #*P*<0.05 compared to the FA group. **C.** Renal level of caspase3 in different groups. (Mean ± SEM, N = 6), *P<0.05 compared to the control and group, ##*P*<0.01 compared to the FA group, and #*P*<0.05 compared to the FA group (one-way ANOVA followed by Tukey's *post hoc* test)

FA: Folic acid; RA: Rosmarinic acid

P53 gene expression significantly increased in the FA group compared to the control group (4.99±1.17 vs 1±0; *P*<0.001). However, when treated with RA, there was a significant decrease in p53 tissue levels in both groups (1.54±0.052 and 0.80±0.091 vs 1±0; *P*<0.01 and *P*<0.001, respectively)(Figure 3A).

As presented in Figure 3B, the ratio of Bax/Bcl-2 expression in the renal tissue significantly increased in the FA group compared to the control group (1.40±0.16 vs 1; *P*<0.05). However, after treatment with 50 and 100 mg/kg RA, their levels significantly lowered compared to the control group (0.66±0.052 and 0.97±0.074 vs 1.40±0.16; *P*<0.01 and *P*<0.05, respectively).

In the same way, the tissue level of caspase-3 was significantly higher in the FA group compared to the control group (4.83±0.023 vs 3.55±0.02; *P*<0.05); and a remarkable decrement was seen after RA treatment in both treated groups compared to the FA group (2.35±0.13 and 3.7±0.12 vs 4.83±0.023; *P*<0.01 and *P*<0.05, respectively) (Figure 3C). 

### Effects of RA on renal tissue inflammation in mice induced by FA

**Figure 4:**
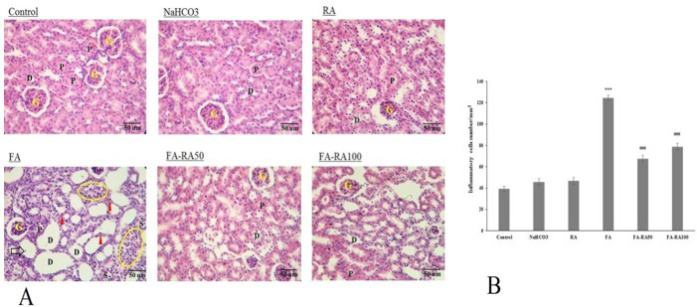
**A**. Results of hematoxylin and eosin (H&E) staining in different groups of mice induced by folic acid G: glomerulus; D: distal tubules; P: proximal tubules; S: swollen tubular cells; red arrowheads: pyknotic nuclei; Black arrows: detached tubular cells; oval circle, infiltrations of mononuclear cells, (Magnification X400). **B.** Numbers of inflammatory cells in different groups. (Mean ± SEM, N = 6), ***P<0.001 compared to the control, NaHCO_3_, and RA groups, ###*P*<0.01 compared to the FA group, and ###*P*<0.001 compared to the FA group (one-way ANOVA followed by Tukey's *post hoc* test)

Figure 4 showed that inflammatory cells in the renal tissue significantly increased in the FA group compared to the control group (124.6±2.5 vs 39.2±2.59; *P*<0.001), and as expected, after treatment with 50 and 100 mg/kg RA, the levels significantly decreased compared to the FA group (67.6±2.85 and 78.8±3 vs 124.6±2.5; *P*<0.001). Degenerative changes, detached tubular cells, and mononuclear cell infiltration were also observed after FA injection, which were improved in the FA-RA50 and FA-RA100 groups. 

### Effects of RA on renal BUN and creatinine (Cr) in mice induced by FA

**Figure 5:**
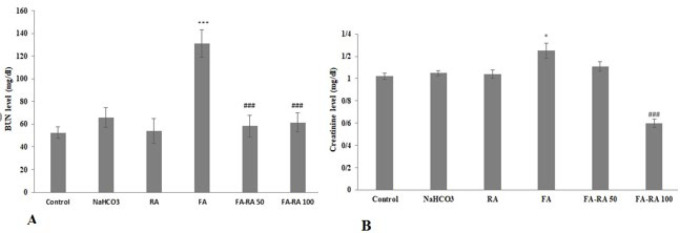
**A.**BUN level in different groups in different groups of mice induced by folic acid (Mean ± SEM, N = 6), ***P<0.001 compared to the control, NaHCO_3_ and RA groups, ###*P*<0.001 compared to the FA group. **B.** Creatinine levels in different groups. (Mean ± SEM, N = 6), *P<0.05 compared to the control and NaHCO_3_ group, ###*P*<0.001 compared to the FA group (one-way ANOVA followed by Tukey's *post hoc* test)

BUN: Blood urea nitrogen; RA: Rosmarinic acid; FA: Folic acid

Results showed that Blood urea nitrogen (BUN) significantly increased in the FA group compared to the control group (*P*<0.001); however, its level decreased after RA treatment in both treated groups (*P*<0.001) (Figure 5A). In the same way, blood creatinine (Cr) levels showed a marked increased level compared to the control group (*P*<0.05). Meanwhile, Cr level significantly declined in the FA-RA100 (*P*<0.001) (Figure 5B).

## Discussion

The current study showed that RA effectively improves inflammation and reduces apoptosis in the renal damage of C57/BL6 mice. To our knowledge, no research has been reported in this regard. The pharmacological effects of RA are diverse and include anti-inflammatory, anti-oxidative, anti-apoptotic, and anti-tumorigenic properties (29,30). So far, extensive *in vitro* and *in vivo* studies of inflammatory diseases, including atopic dermatitis, colitis, arthritis, and allergy, have demonstrated the anti-inflammatory properties of RA (31-33). 

Inflammation is a crucial aspect of innate immunity and helps the immune system’s homeostasis, but excessive inflammation can result in chronic or systemic inflammatory disorders. Liu *et al*.’s findings indicate that NF-κB transcription factor plays a major role in inducing inflammation, apoptosis, and expression of proliferative genes responsible for tissue repair and regeneration (34). FoxO3 negatively regulates NF-κB signaling, and it has been demonstrated that overexpression of FoxO3 suppresses NF-κB activity (35). It has also been shown that FoxO3 activation reduces inflammation by influencing NLRP3 (36). Furthermore, FoxO3 regulates several processes, including cell cycle, DNA repair, hypoxia, apoptosis, autophagy, etc (37,38). FoxO3 regulates the transcription of genes related to autophagy and stops apoptosis by inhibiting caspase-3 (39,40). TNFα and IL6 are also key factors in regulating the cytokine cascade and inflammatory diseases that can be induced by NF-κB (41). Besides, activated FoxO3 decreases IL6 levels via decrement of gene expression and protects against renal fibrosis (18). In line with the studies mentioned above, the current research results showed that RA treatment increased the expression of FoxO3 and decreased the expression of NFκB and TNFα in the kidney tissue. Microscopic analyses also showed that the number of inflammatory cells in the renal tissue was significantly reduced in the groups treated with RA, and these results could confirm the positive effects of RA administration on decrement in NF-κB, TNFα, and IL6. Other researchers have also reported that the anti-inflammatory effects of RA were performed through the modulation of NF-κB and metalloproteinase-9 (MMP9) (42). A systematic review has also documented that most anti-inflammatory trials of RA have focused on paw edema, acute liver injury, and asthma, producing the greatest results (43). In a similar study, RA ameliorated cadmium-induced renal injury by modulating the NF-κB/TNFR2/MAPK/PKC-δ pathway (44). By suppressing the production of NF-κB and TNF-α, Dumitrovich *et al*. have also reported the nephroprotective activities of RA against cisplatin-induced renal injury in animal experiments (45). In the same way, RA significantly reduced IL-6 levels (46). Compatible with other studies, it seems that RA can increase FoxO3 protein expression and inhibit NF-κB production, and thereby, expressions of TNFα and IL6 decrease, and inflammation attenuates.

Inflammation and homeostasis imbalance can lead to cellular death and organ damage (47). Therefore, cell injury and apoptosis will occur following the increase of inflammatory factors in kidney failure (48). Apoptosis is a controlled type of cell death that merely removes undesirable cells or structures that may be damaged, unnecessary, or redundant (49,50). P53 protein, as an upstream protein, can regulate the expression of many genes in apoptosis and plays an important role in apoptosis and many vital functions of the cell (51). Also, nuclear transcription factor (NF-κB) and Bax are apoptosis activators (52). On the other hand, extensive research on the inflammatory response in renal disorders has shown that increased pro-inflammatory cytokines such as IL-6 and TNF-α can promote apoptosis (53, 54).

The anti-apoptotic properties of Bcl-2 seem to be associated with its heterodimerization with the proapoptotic Bax protein. Thus, the relationship between Bcl-2 and Bax may determine a sensitivity to apoptosis (55). Reduced levels of caspase-3, which is thought to be the last functioning protein in the apoptotic process, can be a sign that apoptosis is being inhibited (56). overexpression of FoxO3 also reverses apoptosis through genetical processes and down-regulation of caspase-3 and P53 genes (57). In this regard, ALTamimi *et al*. have reported that curcumin reversed diabetic nephropathy in rats through activation of FoxO3 and reduction of NF-κB and oxidative stress (58). Our findings showed that RA administration decreased P53, Bax/Bcl-2 ratio, and Caspase-3 as the main indicators of apoptosis in RA-FA 50 and RA-FA 100 groups compared to the FA group. In confirmation of the above findings, a study reported that via stimulating the PI3K/Akt signaling pathway, RA reduces the activities of NOXs, removes reactive oxygen species (ROS), prevents oxidative damage and apoptosis, and ultimately preserves lung structure and function in cases of pulmonary ischemia-reperfusion injury (59). Another study reported that RA protects C2C12 myoblast cells from H_2_O_2_-induced damage by protecting DNA damage caused by oxidative stress and apoptosis (60). Also, in renal injury induced by cisplatin, RA demonstrated anti-apoptotic action by reducing P53, phosphorylated P53, and active Caspase-3 expression (45). Our results, consistent with the previous studies, indicate that treatment with RA led to NF-κB level decrement (due to FoxO3 overexpression), diminished Bax/Bcl-2 ratio, and P53 expressions, thus decreasing Caspese3 level. 

Finally, biochemical and histological findings confirmed the above data and clearly showed that renal injury was ameliorated after RA administration. Several documents demonstrate the positive effects of RA on renal injuries (26, 27).

**Table 1:** These primers were used for real-time reverse transcription polymerase chain reaction (RT-PCR) assay

**Gene**	**Forward**	**Reverse**
**Bax**	AGACAGGGGCCTTTTTGCT	AATTCGCCGGAGACACTCG
**Bcl-2**	CTTTGAGTTCGGTGGGGTCA	AGTTCCACAAAGGCATCCCA
**IL6**	TCTGAAGGACTCTGGCTTTG	GATGGATGCTACCAAACTGGA
**TNFα**	AGGGTCTGGGCCATAGAACT	CCACCACGCTCTTCTGTCTAC
**P53**	GCCATGGCCATCTACAAGAA	CTCGGGTGGCTCATAAGGTA
**GAPDH**	TGGCCTTCCGTGTTCCTAC	GAGTTGCTGTTGAAGTCGCA

RA: Rosmarinic acid; FA: Folic acid

FA: Folic acid

## Conclusion

Overall, the current study demonstrated that RA plays a notable role in improving inflammation and apoptosis in folic acid-induced renal failure, which can result from its involvement in the FoxO3/NF-κB signaling pathway.

## Declaration

We have not used any AI tools or technologies to prepare this manuscript.

## Limitations of the Study

This study’s limitations include animal model induction and the high cost of kits and materials.

## Financial Source

There was no financial source for this study.
